# Kabasura Kudineer (KSK), a poly-herbal Siddha medicine, reduced SARS-CoV-2 viral load in asymptomatic COVID-19 individuals as compared to vitamin C and zinc supplementation: findings from a prospective, exploratory, open-labeled, comparative, randomized controlled trial, Tamil Nadu, India

**DOI:** 10.1186/s13063-021-05583-0

**Published:** 2021-09-15

**Authors:** S. Natarajan, C. Anbarasi, P. Sathiyarajeswaran, P. Manickam, S. Geetha, R. Kathiravan, P. Prathiba, M. Pitchiahkumar, P. Parthiban, K. Kanakavalli, P. Balaji

**Affiliations:** 1 Siddha Central Research Institute, Chennai, India; 2 ICMR-National Institute of Epidemiology, Chennai, India; 3 Government Stanley Medical College, Chennai, India; 4 State Licensing Authority (Indian Medicine), Chennai, India; 5 Department of Indian Medicine and Homeopathy, Govt. of Tamil Nadu, Chennai, India; 6 Central Council for Research in Siddha, Ministry of AYUSH, Chennai, India

**Keywords:** Siddha medicine, Polyherbal decoction, Asymptomatic COVID-19 cases, Kabasura Kudineer, AYUSH, Traditional medicine

## Abstract

**Introduction:**

Despite several ongoing efforts in biomedicine and traditional medicine, there are no drugs or vaccines for coronavirus disease 2019 (COVID-19) as of May 2020; Kabasura Kudineer (KSK), a polyherbal formulation from India’s Siddha system of medicine, has been traditionally used for clinical presentations similar to that of COVID-19. We explored the efficacy of KSK in reducing viral load and preventing the disease progression in asymptomatic, COVID-19 cases.

**Methods:**

A prospective, single-center, open-labeled, randomized, controlled trial was conducted in a COVID Care Centre in Chennai, India. We recruited reverse-transcription polymerase chain reaction (RT-PCR)-confirmed COVID-19 of 18 to 55 years of age, without clinical symptoms and co-morbidities. They were randomized (1:1 ratio) to KSK (60 mL twice daily for 7 days) or standard of care (7 days supplementation of vitamin C 60,000 IU morning daily and zinc 100 mg evening daily) groups. The primary outcomes were reduction in the SARS-CoV-2 load [as measured by cyclic threshold (CT) value of RT-PCR], prevention of progression of asymptomatic to symptomatic state, and changes in the immunity markers including interleukins (IL-6, IL-10, IL-2), interferon gamma (IFNγ), and tumor necrosis factor (TNF α). Siddha clinical assessment and the occurrence of adverse effects were documented as secondary outcomes. Paired *t*-test was used in statistical analysis.

**Results:**

Viral load in terms of the CT value (RdRp: 95% CI = 1.89 to 5.74) declined significantly on the seventh day in the KSK group and that of the control group, more pronounced in the study group. None progressed to the symptomatic state. There was no significant difference in the biochemical parameters. We did not observe any changes in the Siddha-based clinical examination and adverse events in both groups.

**Conclusion:**

KSK significantly reduced SARS-CoV-2 viral load among asymptomatic COVID-19 cases and did not record any adverse effect, indicating the use of KSK in the strategy against COVID-19. Larger, multi-centric trials can strengthen the current findings.

**Trial registration:**

Clinical Trial Registry of India CTRI2020/05/025215. Registered on 16 May 2020

## Background

Severe acute respiratory syndrome coronavirus 2 (SARS-CoV-2) infection causing coronavirus disease 2019 (COVID-19) has affected more than 190 million people and caused 408,600 deaths globally as of 20th July 2021 [1]. Clinical symptoms of COVID-19 include mild illness (upper respiratory tract infections, fever, anorexia, malaise, muscle pain, sore throat, dyspnea, nasal congestion, headache), pneumonia, severe pneumonia, acute respiratory failure, sepsis, and septic shock [2]. Confirmatory diagnosis of COVID-19 is done through real-time reverse transcription-polymerase chain reaction assay (RT-PCR) [3]. People who are COVID-19-positive but do not exhibit any of the symptoms are termed asymptomatic [4]. These asymptomatic COVID-19 individuals could contribute to the rapid and extensive spread of SARS-CoV-2 [5]. The prevalence of asymptomatic individuals among SARS-CoV-2-infected people is about 40–45% [6].

Despite intensive ongoing efforts at drug discovery and vaccine development, there are no proven interventions, as of May 2020 [7]. Due to the novel nature of the viral infection, the trials have explored drugs/formulations from both modern and traditional medical systems [8]. In the Indian context, Ayurveda, yoga and naturopathy, Unani, Siddha, Sowa-Rigpa, and homoeopathy (collectively called as AYUSH) are traditional medical systems recognized and patronized by the government under the Indian Ministry of AYUSH. Of the AYUSH systems, Ayurveda, yoga, and Siddha systems of medicine are indigenous to India. While Ayurveda has a wider presence, the Siddha system of medicine is rooted in the southern Indian state of Tamil Nadu and is practiced in ethnic Tamil populations of the world.

The Siddha system of medicine defines *Uyirthathukkal* (three humors, namely *Vali* (*Vatham*), *Azhal* (*Pitham*), and *Iyam* (*Kabam*)) and *Udalkattugal* (*Saaram*, *Senneer*, *Oon*, *Kozhuppu*, *Enbu*, *Moolai*, *Sukkilam/Suronitham*) as fundamental principles of the human body and also conceptualizes unique individual body constitution of every person called as *Udaliyal* [9]. These traditional systems play a vital role in the management of national health care needs [10]. They advocate drugs of herbal, mineral, and animal origins for treating various diseases. Almost 360 thousand traditional formulations/practices of Ayurveda, Siddha, Unani, and Sowa-Rigpa have been transcribed into the Traditional Knowledge Digital Library in India [11]. The reported action of herbal drugs depends on the phytochemical components [12]. Non-specific targeting antiviral therapeutic methods triggered the advancement in research on plant-based antiviral agents. Herbal antiviral formulations have contributed to managing viral infections [13]. It was found after the screening of hundreds of Chinese medicinal herbs, extracts from *Lycoris radiata*, *Artemisia annua*, *Pyrrosia lingua*, and *Lindera aggregata* had anti-SARS-CoV effect [14].

In the context of COVID-19, it is important to revisit the research potential of such antiviral herbal formulations. In fact, one such Siddha classical poly-herbal formulation *Nilavembu kudineer* (made as a decoction) documented antiviral action against the dengue virus [15]. Herbal formulations from the Siddha system of medicine have been widely used during the times of outbreaks of dengue in Tamil Nadu, and specifically, *Nilvembu kudineer* is reported to have contributed to the reduction in severe outcomes due to chikungunya and dengue in Tamil Nadu during 2015 [16]. Further, many such formulations from the Siddha system of medicine have immune-stimulating and inflammation-modulating effects [17]. Hence, on the basis of such documentation in the past, an Indian advisory by the Ministry of AYUSH incorporated the role of the Siddha system of medicine for COVID-19 [18]. On the lines of the national advisory, the state of Tamil Nadu proposed the use of Siddha formulations in the management of asymptomatic and mild COVID-19 through a new scheme, called *Arokkiam* in which *Kabasura Kudineer* is one such polyherbal Siddha formulation [19].

*Kabasura Kudineer* (KSK) is indicated for use in *Aiya suram* (fever) and *Aiya noigal* (respiratory diseases) in the Siddha system of medicine. KSK consists of 15 herbal ingredients of which five are shown to possess antiviral activity [20]. Cucurbitacin B (− 112.09), cardiofoliolide (− 111.5), apigenin (− 98.84), and pyrethrin (− 92.98) present in the herbal decoction were found to be effective in preventing SARS-CoV-2 binding and replication [21]. Standardization of KSK has documented organoleptic characteristics, physicochemical values, thin layer chromatographic profiles, and high-performance TLC fingerprints [22]. Furthermore, toxicological studies have documented the safety of KSK [23]. KSK possesses anti-pyretic, anti-inflammatory, and anti-bacterial effects [23]. In the absence of any systematic clinical documentation of the effectiveness of KSK for COVID-19, we proposed a prospective, exploratory, randomized, controlled trial on asymptomatic COVID-19. The cycle threshold value of RT-PCR, symptoms assessment, and cytokines have been explored as outcome measures in the effectiveness studies on asymptomatic COVID-19 cases earlier [5].

In view of the above, the primary objective of the trial was to evaluate the efficacy of KSK in terms of reduction in viral load, prevention of progression of asymptomatic to symptomatic status, and any changes in the immunological markers. The secondary objectives were to assess the symptoms based on the standard Siddha *Udaliyal* protocol and document the adverse effects of KSK.

## Methods

### Study design

This was a single-center, open-labeled, randomized controlled trial in a parallel group with a 1:1 allocation ratio [24].

### Cases

As per the state protocol [25], the health authorities identified high-risk individuals through *contact tracing* of indexed cases and screened for SARS-CoV-2 using the RT-PCR method, at the state-run COVID-19 testing centers in the capital city of Chennai, Tamil Nadu. The COVID-19-confirmed cases, without any symptoms were referred to the COVID Care Centre managed by the Government Stanley Medical College, Chennai. We recruited all asymptomatic COVID-19-positive cases aged 18 to 55 years for the trial and randomly assigned them to the control or study groups. We excluded those with diabetes, hypertension, bronchial asthma, and malignancy. Pregnant and lactating women and those participating in other COVID-19 trials were excluded.

### Study setting

We did the trial at a non-hospital setting designated as COVID Care Centre and managed by the State Government Stanley Medical College, Chennai.

### Interventions

#### Study group (KSK)

The study drug KSK is a Siddha polyherbal formulation that contains dried parts of 15 herbs in equal proportion (Table 1). The study drug was procured in the *chooranam* (powder) form, as a single batch for maintaining homogeneity, from the public sector company Tamil Nadu Medicinal Plants Corporation Limited (TAMPCOL). TAMPCOL is a Good Manufacturing Practices (GMP)-certified company run by the Government of Tamil Nadu, India. KSK *Chooranam* (powder) was manufactured as per the National Siddha Formulary of India (part I, 1st edition 1992) [26] and supplied by TAMPCOL along with a quality control certificate. A decoction was prepared by boiling 5 g of KSK *Chooranam* in 240 mL water and reduced to one-fourth (60 mL) and filtered through a sieve adopting the traditional method of decoction preparation. A dose of 60 mL decoction was given to the participants after food, orally in the morning and evening for 7 days.

**Table 1:** Composition of *Kabasura Kudineer* Siddha polyherbal preparation

S. no.	Botanical name of the ingredients	Siddha name	Botanical family	Part used	Parts
1.	*Zingiber officinale* Rosc.	*Chukku*	Zingiberaceae	Rhizome	1 part
2.	*Piper longum* L.	*Thippili*	Piperaceae	Fruit	1 part
3.	*Syzygium aromaticum* (L.) Merr. & Perry	*Kirambu*	Myrtaceae	Flower bud	1 part
4.	*Anacyclus pyrethrum* (L.) Lag.	*Akkirakaram*	Asteraceae	Root	1 part
5.	*Tragia involucrata* L.	*Konchori*	Euphorbiaceae	Root	1 part
6.	*Hygrophila auriculata* (Schum.) Heine Lam.	*Neermulli*	Acanthaceae	Root	1 part
7.	*Terminalia chebula* Retz.	*Kadukkai*	Combretaceae	Fruit rind	1 part
8.	*Justicia adhatoda* L.	*Adathodai*	Acanthaceae	Leaves	1 part
9.	*Plectranthus amboinicus* (Lour.) Spreng.	*Karpooravalli*	Lamiaceae	Leaves	1 part
10.	*Costus speciosus* (J. Koenig) Sm.	*Kostam*	Costaceae	Root	1 part
11.	*Tinospora sinensis* (Lour.) Merr.	*Seenthil*	Menispermaceae	Stem	1 part
12.	*Clerodendrum serratum* (L.) Moon	*Siruthekku*	Verbanaceae	Root	1 part
13.	*Andrographis paniculata* (Burm.f.) ex Nees	*Nilavembu*	Acanthaceae	Whole plant	1 part
14.	*Cyperus rotundus* L.	*Koraikizhangu*	Cyperaceae	Root tuber	1 part
15.	*Cissampelos pareira* L.	*Vattathiruppi*	Malvaceae	Root	1 part

#### Control group (CZ)

The patients in the control group received vitamin C (60,000 IU) and zinc tablets (100 mg) orally in the morning and evening, respectively, for 7 days.

### Outcome measures

The primary outcomes were the reduction in the SARS-CoV-2 load [as measured by CT value of RT-PCR] from the baseline to seventh day of treatment, preventing the progression of asymptomatic to symptomatic state (clinical symptoms like fever, cough, and breathlessness) and changes in the immunity markers [interleukins (IL)-6, IL-10, IL-2; interferon gamma (IFNγ); and tumor necrosis factor (TNF) alpha]. COVID-19 symptoms as per standard Siddha *Udaliyal* assessment protocol and the occurrence of adverse effects were documented as secondary outcomes.

Clinical assessments were carried out during the entire treatment period. These included examination based on the principles of the Siddha system of medicine viz., *Siddha Udaliyal* (body constitution/physique) [27], *Uyir thathukkal* [*tridosha*] [*Vali -* (*Uyirkkal* (*Vayu* responsible for respiration), *Paruvukal* (*Vayu* responsible for circulation), *Kizhnokkukkal* (*Vayu* responsible for downward movements), *Nadukkal* (*Vayu* responsible for homeostasis), *Melnokkukal* (*Vayu* responsible for upward movements), *Nagan* (*Vayu* responsible for intellectual functions), *Koorman* (*Vayu* responsible for ophthalmic function), *Kirukaran* (*Vayu* responsible for secretory functions), *Devathathan* (*Vayu* responsible for fatigue), *Thananjeyan* (intracranial *Vayu*), *Azhal* (*Aakkanal* (*Thee* responsible of digestion), *Vannayeri* (*Thee* responsible of color of blood), *Aattralangi* (*Thee* responsible of cardiac functions), *Olloli Thee* (*Thee* responsible of lusture of skin), *Nokkazhal* (*Thee* responsible of vision), *Iyam* (*Aliiyam* (*Iyam* responsible for innate potential), *Neerppiyam* (*Iyam* responsible for digestion), *Suvaikanaiyam* (*Iyam* responsible for taste), *Niraivaiyam* (*Iyam* responsible for strengthen of sense organs), *Onriyaiyam* (*Iyam* responsible for Joint lubrication))] and *Udal thathukkal* (examination of *Saaram* (lymph), *Senneer* (blood), *Oon* (muscle), *Kozhuppu* (fat), *Enbu* (bone), *Moolai* (bone marrow), *Sukkilam/Suronitham* (sexual secretions)) [9]. Laboratory and biochemical assessments of the renal function, liver function, hemogram, inflammatory markers, electrolytes, nasopharyngeal (NP), and oropharyngeal (OP) swabs were done at baseline and on the seventh day. The NP/OP swabs were used for viral RNA detection and quantification of E-gene, RNA-dependent RNA polymerase (RdRp) {specific target for SARS-CoV-2}, and N-gene (CT value; RT PCR). Viral RNA was extracted with the Qiagen Minikit and Machine Qiacube Automated Extraction unit and Amplification, Detection, and Quantification in Rotor-Gene Q Real-Time PCR from Qiagen, Germany, using AllplexTM 2019-nCoV Assay, Seegene Inc., Seoul, Republic of Korea. Thermal cycling using six independently controlled thermal electric modules to maintain tight temperature uniformity (range 0–100 °C with an accuracy of ± 0.2 °C of the programmed target at 90 °C and a uniformity of ± 0.4 °C well to well within 10 s at arrival at 90 °C) at all points during run with efficient optimization. Adverse events were monitored daily. Clinical data was recorded on paper-based case record forms.

### Sample size

We did not have prior data on the efficacy of Siddha medicine in the management of COVID-19. Hence, as an exploratory study of the efficacy of KSK on asymptomatic COVID-19 patients, we decided to recruit 30 participants in each of the two groups.

### Randomization

Eligible participants were randomly assigned (1:1) to either study (KSK) or control group (CZ). The random sequence was prepared using a random number generation in a spreadsheet program by the statistician who was not directly involved in the trial. Thirty sealed opaque envelopes were prepared for each of the study and control groups. Randomization was done by selection of a sealed opaque envelope from a closed box

### Statistical analysis

The primary outcome, SARS-CoV-2 viral load in terms of CT value differences, was assessed for each group. This outcome was reported as a mean difference with 95% confidence interval (CI). A comparison of the mean difference was executed using mean ± standard deviation. The secondary outcomes, safety in terms of biochemical parameters, was described using mean ± standard deviation for normally distributed values and median with its interquartile range for values which do not follow a normal distribution. We proposed descriptive statistics for reporting adverse events.

## Results

**Fig. 1:**
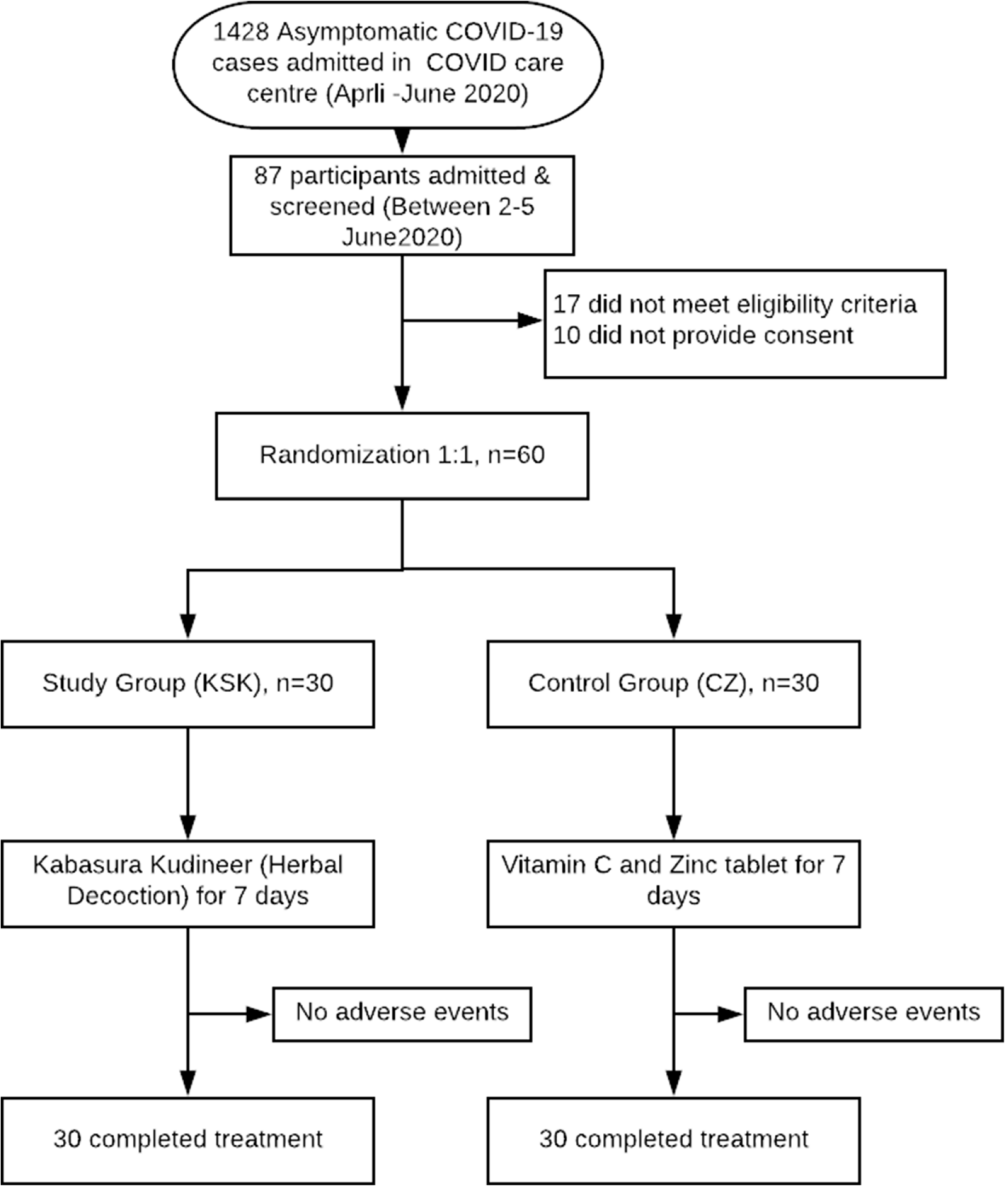
Trial flowchart

As per the state guidelines, asymptomatic cases were admitted in the COVID care center (Government of Tamil Nadu). We used this setting to recruit trial participants. During the recruitment period (2–4 June 2020), a total of 87 participants were admitted in this setting. We screened the 87 and found 60 eligible and consented to participate in the trial (Fig. 1). Except for age and gender, the study and control groups were almost similar in all the baseline characteristics (Table 2). There was no significant difference in the biochemical parameters (Table 3).

**Table 2:** Frequency (#) of baseline characteristics of the study and control groups

Characteristics		Study group (*n* = 30)	Control group (*n* = 30)
Age (in years)	[Mean ± standard deviation (SD)]	36.33 ± 10.54	34.47 ± 11.28
Male gender		25	20
BMI (kg/m^2^)	[Mean ± SD]	24.70 ± 4.64	23.90 ± 3.13
Education	Higher secondary	17	17
	Diploma	4	1
	Degree	9	12
Occupation	Employed	26	20
	Unemployed	0	1
	Homemaker	2	6
	Student	2	3
Exposure history	Contact with COVID-19 individual	11	12
	Do not know	19	18
Body constitution (phenotype) as per Siddha system	*Vali*	15	16
	*Azhal*	9	9
	*Aiyam*	6	5

**Table 3:** Biochemical parameters [mean (SD)] of the study group and control group before and after treatment

Investigation	Study group (*n* = 30)		Control group (*n* = 30)	
	Baseline	End line	Baseline	End line
Renal function test				
BUN (7.0–21 mg/dL)	12.08 (3.96)	9.73 (2.41)	11.68 (3.66)	10.86 (2.14)
Creatinine (0.9–1.3 mg/dL)	0.99 (0.12)	0.89 (0.11)	0.94 (0.18)	0.98 (0.16)
Electrolytes				
Bicarbonate (22–29 mmol/L)	22.19 (0.79)	22.19 (0.50)	22.71 (0.94)	21.97 (0.83)
Chloride (98–107 mmol/L)	97.98 (2.89)	98.43 (2.47)	98.32 (3.36)	98.94 (3.79)
Potassium (3.5–5.1 mmol/L)	4.21 (0.49)	4.51 (0.47)	4.69 (0.51)	4.25 (0.55)
Sodium (136–145 mmol/L)	142.88 (2.8)	140.49 (1.88)	141.59 (3.38)	141.14 (2.66)
Liver function test				
SGOT* (5–40 U/L)	33.05 (25.53–47.70)	27.65 (22.32–33.25)	26 (23.27–30.95)	26.15 (22.55–31.52)
SGPT* (5–41 U/L)	37.75 (22.60–52.15)	29.60 (20.70–41.15)	23 (16–31.92)	27.85 (21.85–37.87)
Alkaline phosphatase (53–128 U/L)	74.26 (17.68)	76.71 (19.63)	89.45 (18.35)	86.68 (21.18)
Albumin (3.5–5.2 gm/dL)	4.34 (0.19)	4.32 (0.19)	4.27 (0.20)	4.31 (0.21)
Globulin (2.3–3.6 gm/dL)	3.33 (0.31)	3.29 (0.27)	3.49 (0.39)	2.71 (0.41)
AG ratio (1.1–2.2)	1.31 (0.16)	1.32 (0.11)	1.23 (0.14)	1.62 (0.21)
Total protein (6.0–8.0 gm/dL)	7.67 (0.31)	7.59 (0.38)	7.77 (0.37)	7.03 (0.44)
GGT* (< 55 U/L)	27.65 (19.33–40.40)	28.05 (22.22–35.60)	27.95 (21.40–41.02)	25 (20.57–43.92)
Total bilirubin (0.1–1.2 mg/dL)	0.81 (0.34)	0.80 (0.33)	0.48 (0.15)	0.50 (0.16)
Bilirubin direct (0.0–0.3 mg/dL)	0.25 (0.07)	0.22 (0.05)	0.16 (0.04)	0.16 (0.03)
Bilirubin indirect (0.1–1.0 mg/dL)	0.55 (0.27)	0.59 (0.27)	0.32 (0.12)	0.33 (0.13)
Blood sugar				
HbA1c	5.93 (1.65)	5.99 (1.66)	6.69 (2.29)	6.68 (2.23)
Inflammatory marker				
CRP* (≤ 6 mg/L)	3.97 (1.6–6.5)	1.60 (1.0–5.32)	4.05 (1.45–7.4)	2 (1.15–4.25)
Complete blood count				
Total RBC count (4.2–5.4 mill/cu.mm)	5.14 (0.52)	5.05 (0.49)	4.87 (0.55)	4.67 (0.56)
Total leukocyte count (4000–11,000 cells/cu.mm)	6667.33 (2071)	7277.03 (1419.75)	7000.00 (1866.56)	8167.33 (1629.38)
Neutrophils (40–75%)	57.09 (10.33)	59.50 (6.91)	50.15 (8.40)	52.81 (6.66)
Lymphocytes (20–45%)	33.68 (8.77)	32.26 (6.86)	40.19 (7.45)	38.22 (6.66)
Eosinophils* (01–06%)	1.6 (0.75–2.9)	2.3 (1.10–3.22)	1.7 (0.70–2.9)	1.8 (1.37–2.62)
Basophils (00–02%)	0.4 (0.2)	0.26 (0.14)	0.38 (0.11)	0.15 (0.06)
Monocytes (01–10%)	6.24 (1.78)	5.40 (1.24)	7.09 (1.71)	6.37 (1.24)
Absolute neutrophil count (1.5–6.6 × 10^3^/μL)	3.94 (1.76)	4.32 (1.10)	3.57 (1.30)	4.31 (1.03)
Absolute lymphocyte count (1.5–3.5 × 10^3^/μL)	2.13 (0.54)	2.29 (0.52)	2.76 (0.77)	3.11 (0.83)
Absolute eosinophil count* (0.04–0.44 × 10^3^/μL)	0.11 (0.04–0.16)	0.15 (0.08–0.24)	0.12 (0.04–0.18)	0.15 (0.10–0.23)
Absolute basophil count (< 0.2 × 10^3^/μL)	0.02 (0.01)	0.02 (0.01)	0.02 (0.01)	0.02 (0.01)
Absolute monocyte count (< 1.0 × 10^3^/μL)	0.39 (0.09)	0.38 (0.09)	0.49 (0.18)	0.52 (0.16)
Platelet count (150–450 × 10^3^/μL)	274.53 (80.13)	315.60 (81.41)	298.30 (69.65)	303.33 (86.00)
PCT (0.18–0.28%)	0.27 (0.07)	0.31 (0.07)	0.29 (0.06)	0.28 (0.07)
MPV (8.0–13.3 fL)	10.14 (0.99)	10.41 (1.18)	10.12 (1.14)	9.38 (0.74)
Hemoglobin (12.5–16.0 g/dL)	14.54 (1.56)	14.37 (1.47)	13.61 (1.85)	13.43 (1.84)
MCH (27–32 pg)	28.38 (2.95)	28.57 (2.74)	27.98 (3.33)	28.84 (3.49)
MCHC (32–36 g/dL)	32.75 (1.56)	33.79 (0.98)	32.85 (1.32)	34.71 (1.18)
MCV (78–100 fL)	86.49 (6.48)	84.43 (6.63)	85.02 (7.85)	82.98 (8.16)
PCV (37–47%)	44.33 (3.83)	42.47 (3.82)	41.33 (4.90)	38.66 (4.75)
RDW-CV (11.5–16.0 %)	14.09 (1.72)	13.24 (1.57)	14.13 (1.83)	13.34 (1.96)
RDW-SD (39–46 fL)	42.4 (3.46)	39.05 (4.12)	41.86 (5.53)	38.50 (5.27)

*Note*: Data expressed in mean (SD); *data expressed as median (IQR)

*BUN* blood urea nitrogen, *SGOT* serum glutamic oxaloacetic transaminase, *SGPT* serum glutamic pyruvic transaminase, *AG ratio* albumin/globulin ratio, *GGT* gamma glutamyl transpeptidase, *CRP* C-reactive protein, *PCT* plateletcrit, *MPV* mean platelet value, *MCH* mean corpuscular hemoglobin, *MCHC* mean corpuscular hemoglobin concentration, *MCV* mean corpuscular volume, *PCV* packed cell volume, *RDW-CV* red blood cell distribution width coefficient variant, *RDW-SD* red blood cell distribution width standard deviation

All the patients were asymptomatic in both groups at the baseline, and no one progressed to the symptomatic state at the end of the treatment. Viral load (as per CT value for RdRp gene) declined significantly on the seventh day in the study group as well as in the control group (Table 4). The mean difference in the CT value was more pronounced in the study group.

**Table 4:** Viral load (cyclic threshold value; mean) for the study group and control group before and after treatment

Group	Type of gene	Baseline CT value	End line CT value	Difference between baseline and end line	95% CI of the difference	
		Mean ± SD	Mean ± SD	Mean	Lower	Upper
Study	E gene	29.22 ± 6.44	33.29 ± 4.45	4.07	2.17	5.97
	RdRp gene	29.63 ± 6.61	33.45 ± 4.14	3.81	1.89	5.74
	N gene	28.63 ± 6.15	33.22 ± 4.54	4.59	2.76	6.41
Control	E gene	26.53 ± 5.82	28.05 ± 4.42	1.52	− 0.60	3.65
	RdRp gene	26.73 ± 5.83	29.34 ± 4.53	2.60	0.44	4.76
	N gene	25.39 ± 5.12	27.78 ± 4.02	2.39	0.51	4.27

Of the immunological markers measured, IFNγ (range = 0–5.5 pg/mL), IL 2 (0–12 pg/mL), and TNF-alpha (0–22 pg/mL) were within normal limits in both the groups at the baseline and after 7 days. With regard to IL-6 among the study group, it was within the normal limits (0–12 pg/mL) for 20 patients while the remaining had elevated levels [mean ± SD] (107.20 ± 86.90) at baseline, and after 7 days, two participants had elevated levels [14.35 ± 2.40]. In the control group, all the patients had their IL-6 level within the normal range at the baseline and after 7 days except one (37.91 pg/mL). IL-10 levels in the study group were within the normal limits (0–5 pg/mL) in 19 patients [15.32 ± 4.39] at the baseline, whereas on the seventh day, 25 patients had an increased level [15.32 ± 4.39]. In the control group, 28 patients had an increased level of IL-10 [14.82 ± 3.18] at the baseline, and on the seventh day, 13 patients had elevated levels [13.55 ± 2.77].

In the study group, increased levels of IL-10 (*n* = 7) and IL-6 (*n* = 5) at baseline correlated with that of *Vali* phenotype according to the Siddha system of medicine. We did not observe any changes in the Siddha-based clinical examination parameters*.* There were no adverse events reported in both groups.

## Discussion

We did an exploratory trial on COVID-19-confirmed asymptomatic individuals by comparing the standard of care of vitamin C/zinc supplementation with that of *Kabasura kudineer*, a polyherbal decoction from India’s traditional Siddha system of medicine. We found that KSK significantly reduced the viral load of SARS-CoV-2 after 7 days, when compared to the CZ group. In both groups, the asymptomatic did not progress to the symptomatic state. Additionally, there were no adverse events during the study period.

The anti-SARS-CoV-2 activity of KSK could be due to several reasons. As such, the Siddha system of medicine had been in use for infectious diseases particularly for treating *ammai noi* (*pox viral infections*) and *akki noi* (herpes infections) diseases for more than thousands of years [28]. The herbs as indicated in several Siddha medicine-based formulations have been documented to have multiple effects including that of antiviral activity [29]. Some of these herbal constituents work directly on the therapeutic targets, while others potentially enhance the bioavailability or counteract drug toxicity of the medicine. The constituents of KSK have been documented to have antiviral activity [15, 30–32]. In fact, the most recent in silico study documented the potential in the prevention and replication of SARS-CoV-2 [21]. Further, the antioxidant activity of the herbs could be another explanation for the viral reduction. Increased oxidative stress due to free radicals helps SARS-CoV-2 in replicating and producing inflammatory responses. KSK exhibited strong antioxidant activity in terms of radical scavenging potential against DPPH (IC-50, 9.29 mg/L) [33]. Vitamin C, a documented antioxidant, thus could have played the same role in the COVID-19 management [34].

In addition to antiviral activity, we documented the changes in the immunological markers. KSK might enhance the host antiviral immune responses or block the viral immune escape mechanisms, which in turn shows antiviral activity through immunoregulatory mechanisms. Phytochemicals such as flavonoids, polysaccharides, lactones, alkaloids, diterpenoids, and glycosides present in the herbs present in the KSK have been identified as immunomodulating agents [35]. Immunomodulatory actions of one such ingredient, *Andrographis paniculata* (Nilavembu), has in fact through in-vitro studies showed a reduction of IL-12, TNF alpha, PGE2, NO, COX-2, and iNOS in the microglia and macrophages [35]; inhibition of the production of ROS in neutrophils [36]; regulation of the production of NK cells, IFN gamma, IL-2, and TNF alpha [37]; inhibition of IL-2, IFN gamma and IL-6; reduction of IL-13, IL-4, IL-5, and Th2 cytokines in ovalbumin-induced asthma rat model [38]; and inhibition of Nf-kB binding to DNA [39]. Further, the alkaloid, andrographolide, present in *Nilavembu* has been documented to have diverse biological activities such as increasing cytotoxic potential of lymphocytes, decreasing the cellular and humoral adaptive immune reaction in T cells, antigen-presenting potential of dendritic cells to T cells, and pro-inflammatory proteins expression like iNOS and COX 2 [40–42]. Andrographolide can modulate the innate immune response and regulate the production of antibodies. Another constituent herb, *Tinospora cordifolia* (*Seenthil*), contains cordifolioside A, cordifolioside B, syringin [43], and d-glucan [44] as the main constituents, and they are having immunomodulatory activity. Evidence from in vitro study indicates that dry ginger (*Zingiber officinale*) has immunomodulatory effects and is an effective antimicrobial and antiviral agent [33]. Other ingredients, *Adathoda vasica*, *Costus speciosus*, *Clerodendrum serratum*, and *Anacyclus pyrethrum*, improve the humoral and cellular immunity by immunostimulatory activity [45–48], whereas *Cyperus rotundus* (*Koraikizhangu*) has been shown to modulate both cell-mediated and antibody-mediated immune responses in Wistar rats [49]. In mouse model, water extract of another herb in KSK, *Syzygium aromaticum* (*Kirambu*), inhibited macrophages to produce both IL-1beta and IL-6 [30].

The contribution of asymptomatic COVID-19 to the ongoing transmission has been documented elsewhere [6], though the magnitude is not well characterized. Contact tracing of a COVID-19-confirmed individual is a key strategy to identify asymptomatic COVID-19 individuals. Hence, as a policy, such positive individuals are advised for home quarantine. However, in a country like India, due to socio-demographic and economic reasons, the practice of strict home quarantine is difficult. Hence, in such situations with the limited evidence available from this trial, KSK, which can be prepared and consumed at the household level, seems to be a feasible option in reducing the risk of transmission for the home-quarantined COVID-19 asymptomatic individuals. However, the self-reporting of adverse events has to be insisted with high priority.

Further, clinical features and immunological assessment of asymptomatic patients have not been explored in the literature. Published studies have documented a sub-optimal immune system among asymptomatic COVID-19 individuals in terms of low levels of circulating cytokines with minimal inflammatory responses [50]. Moreover, the reported risk of death among asymptomatic patients might be due to the unidentified levels of cytokines [5]. However, such linkage needs evaluation. As such, there were no reports from India about the immunological assessment of asymptomatic patients among those admitted in COVID care centers. In the trial, we documented elevated levels of cytokines (IL-6) in the study group at baseline, and subsequent to 7 days of administration of KSK, the levels returned back to the normal limits. We could not ascertain the reasons for the different levels of cytokines in the study and control groups. Immunological findings in this study were inconclusive. This finding, nevertheless, warrants further investigation.

In the context of the Siddha system-based examination, we identified that a particular phenotype (*Vali Udaliyal*) individuals had elevated levels of IL-10 and IL-6 in both the study and control groups. This finding correlates well with the Siddha principle of lowered innate immunity among individuals with *Vali* body type [9, 51]. As all cases were asymptomatic and did not progress to the symptomatic stage, as per the Siddha fundamentals, there was no derangement of *Uyrithathukkal* and *udal thathugal*. In fact, all the Siddha parameters were within normal levels.

The reduction in viral load of SARS-CoV-2 in the KSK arm as compared to the CZ group merits discussion. RT-PCR is meant for the detection of SARS-CoV-2 viral genome in biological samples. In the public health context strategy of testing, contact tracing, and isolation, a positive RT-PCR result helps in the isolation of the COVID-19-confirmed individuals and thereby reducing chances of further transmission. CT value of RT-PCR is inversely proportional to the amount of genetic material in the biological sample. In this study, the mean CT value observed in the KSK group at the end line was 33.45 (specifically RdRp). Such a higher CT value indicates lower viral load and proportionately presumed to have reduced infectiousness. The Indian Council of Medical Research advisory stated that the CT value cutoff is 35 for RT-PCR assays on SARS-CoV-2 [52]. An increase of one unit CT value is supposed to reduce infectivity by 32% [53]. Therefore, a higher mean CT value observed at the end line in the intervention group indicates less viral load and thus indicative of lowered transmission potential.

### Limitations

Our study had few limitations. As an exploratory study, findings are mostly hypothesis-generating in nature and warrant further studies. However, despite the small sample size, the comparative nature of the trial and randomization of interventions were useful in documenting the reduction in viral load over and above the decline that could be attributable to the natural decline of SARS-CoV-2 infection. Nevertheless, there were no clinical differences in both groups despite the difference documented in terms of the end line CT values. In addition, in the absence of a follow-up beyond 7 days of treatment, we do not have information on long-term direct or indirect benefits attributable to the consumption of KSK. Further studies with a larger sample size conducted in multiple centers will strengthen the findings of the current study.

## Conclusion

The current exploratory study shows that KSK significantly reduces the SARS-CoV-2 viral load among confirmed asymptomatic COVID-19 cases, when compared to those who received standard of care. Further studies on KSK will explore the public health potential of Siddha medicines in this current pandemic.

## Data Availability

The datasets of this clinical study are available from the corresponding author on reasonable request.
